# Other Studies Show Aspirin Use Is High

**DOI:** 10.1371/journal.pmed.0030110

**Published:** 2006-02-28

**Authors:** Jonathan Brown

**Affiliations:** **1**Kaiser Permanente Center for Health ResearchPortland, OregonUnited States of America

The importance of prophylactic aspirin use in both developed and developing countries can hardly be overemphasized. I am troubled, however, by Stafford and colleagues' failure to cite and discuss United States studies that show much higher rates of antithrombotic use than they report [ 1]. Using a cross-sectional postal survey of 2,500 Kaiser Permanente members with atherosclerotic cardiovascular disease, Brown et al. [ 2] found that 84% were currently taking prophylactic aspirin (72%) or a prescription agent (12%, usually warfarin) in 1999. In an earlier study of another nonprofit-integrated US medical care system, HealthPartners, O'Connor et al. [ 3] similarly found that 71% of members with clinically diagnosed coronary heart disease were taking aspirin. These results are almost triple the approximately 25% that Stafford et al. now report for the 1999–2000 time period (the low point in their time series), and about double the 34% that Stafford [4] previously reported from the National Ambulatory Medical Care Study for patients with coronary heart disease.

Two factors probably account for these differences. First, Stafford et al.'s federal surveys of ambulatory care encounters miss a significant proportion of aspirin use. Studies in a variety of populations that used direct patient surveys and other methods, some of which are cited in Brown et al. [ 1], have found higher rates of aspirin use in a variety of settings than Stafford et al. have reported over the years. Second, nonprofit-integrated medical care programs emphasize and promote aspirin aggressively and effectively. Their members probably have higher rates of aspirin use than individuals in other US care-delivery settings.

The experience of the nonprofit-integrated systems is also important because it calls into question Stafford et al.'s suggestion, emphasized in PLoS's accompanying synopsis, that direct-to-consumer advertising of statins explains the 1997–2000 dip in aspirin use in their data. Kaiser Permanente and HealthPartners members were equally exposed to direct advertising, but maintained high aspirin use during this period—despite probably also using statins (and angiotensin converting enzyme [ACE] inhibitors) at higher rates than individuals in other US care-delivery settings.

The US nonprofit HMO experience nevertheless reinforces the authors' main conclusion that “aggressive and targeted interventions are needed to enhance provider and patient adherence to consensus guidelines for CVD risk reduction” [ 1]. Aggressive and targeted interventions are exactly what these settings use. Major structural factors such as lack of universal health insurance, fee-for-service rather than population-based orientation, and failure to use comprehensive electronic medical record systems will continue to hamper the US, however. Direct-to-consumer advertising, although symptomatic, pales in importance against these other problems.
